# Genome-Wide Identification and Gene Expression Analysis of Acyl-Activating Enzymes Superfamily in Tomato (*Solanum lycopersicum*) Under Aluminum Stress

**DOI:** 10.3389/fpls.2021.754147

**Published:** 2021-12-02

**Authors:** Jian Feng Jin, Qi Yu He, Peng Fei Li, He Qiang Lou, Wei Wei Chen, Jian Li Yang

**Affiliations:** ^1^State Key Laboratory of Plant Physiology and Biochemistry, College of Life Sciences, Zhejiang University, Hangzhou, China; ^2^State Key Laboratory of Subtropical Silviculture, Zhejiang A & F University, Hangzhou, China; ^3^Research Centre for Plant RNA Signaling and Zhejiang Provincial Key Laboratory for Genetic Improvement and Quality Control of Medicinal Plants, College of Life and Environmental Sciences, Hangzhou Normal University, Hangzhou, China

**Keywords:** AAEs superfamily, abiotic stress, Al stress, carboxylic acid, organic acid, oxalate, tomato

## Abstract

In response to changing environments, plants regulate gene expression and subsequent metabolism to acclimate and survive. A superfamily of acyl-activating enzymes (AAEs) has been observed in every class of creatures on planet. Some of plant *AAE* genes have been identified and functionally characterized to be involved in growth, development, biotic, and abiotic stresses *via* mediating diverse metabolic pathways. However, less information is available about AAEs superfamily in tomato (*Solanum lycopersicum*), the highest value fruit and vegetable crop globally. In this study, we aimed to identify tomato AAEs superfamily and investigate potential functions with respect to aluminum (Al) stress that represents one of the major factors limiting crop productivity on acid soils worldwide. Fifty-three *AAE* genes of tomato were identified and named on the basis of phylogenetic relationships between *Arabidopsis* and tomato. The phylogenetic analysis showed that AAEs could be classified into six clades; however, clade III contains no *AAE* genes of tomato. Synteny analyses revealed tomato vegetable paralogs and *Arabidopsis* orthologs. The RNA-seq and quantitative reverse-transcriptase PCR (qRT-PCR) analysis indicated that 9 out of 53 *AAEs* genes were significantly up- or downregulated by Al stress. Numerous *cis*-acting elements implicated in biotic and abiotic stresses were detected in the promoter regions of *SlAAEs*. As the most abundantly expressed gene in root apex and highly induced by Al, there are many potential STOP1 *cis*-acting elements present in the promoter of *SlAAE3-1*, and its expression in root apex was specific to Al. Finally, transgenic tobacco lines overexpressing *SlAAE3-1* displayed increased tolerance to Al. Altogether, our results pave the way for further studies on the functional characterization of *SlAAE* genes in tomato with a wish of improvement in tomato crop in the future.

## Introduction

Aluminum (Al) toxicity is one of the major limiting factors affecting the crop productivity in acidic soils, which occupy nearly 50% of the potential arable lands of the world (von Uexküll and Mutert, 1995). When soil pH is lower than 5.5, ionic Al, mainly Al^3+^, predominates in soil solution, which is highly toxic to plants. The initial and most visible symptom of Al toxicity is inhibition of root elongation by ravaging cell structure of the root apex and thus limiting the mineral nutrient and water uptake and, consequently, hindering the plant growth and development (Kochian, 1995; Ma, 2007; Ryan et al., 2011; Liu et al., 2014). To adapt to Al-toxic environment, plants have evolved two major types of Al-tolerance mechanisms, namely, external exclusion (preventing Al from entering cells of root apex) and internal tolerance mechanisms (detoxifying Al *via* complexation and sequestration) (Kochian et al., 2004, 2015; Liu et al., 2014). Substantial advances have been made toward elucidating the physiological and molecular mechanisms by which plants cope with Al stress (Kochian et al., 2015; Yang et al., 2019). Recently, it has been shown that metabolic change might play important roles in response to Al stress (Lou et al., 2016a,b; Xian et al., 2020). However, the molecular basis of the role of metabolic alterations in Al stress response still needs further elucidation.

The activation of carboxylic acids provides the precursors for pathways that lead to the metabolism of a diverse variety of metabolites, including lipids, amino acids (aa), sugars, and secondary metabolites. In plants, the acyl-activating enzymes (AAEs) superfamily consists of acyl-coenzyme A synthetases (ACSs), 4-coumarate:coenzyme A ligases (4CLs), luciferases, and non-ribosomal peptide synthetases, which are involved in many primary and secondary metabolic pathways. All members of the AAE family have low sequence similarity to each other but share many highly conserved motifs, such as the AMP-binding domain (Shockey and Browse, 2011). As the members of the *Arabidopsis* AAE superfamily were systematically analyzed and identified, more and more metabolic functions of plant *AAE* superfamily genes have been reported. For example, the *Arabidopsis* peroxisomal-localized *OPCL1* (OPDA-CoA ligase) gene, *At1g20510*, is involved in the biosynthesis of jasmonic acid in *Arabidopsis* (Koo et al., 2006; Kienow et al., 2008). The rice fatty acyl-CoA synthase gene, *OsACOS12*, is involved in regulating lipid metabolism-mediated tapetum-programmed cell death, which ultimately affects the male fertility of rice (Yang et al., 2017). The petunia malonyl-CoA synthase gene, *PhAAE13*, is specifically involved in anthocyanin biosynthesis in flowers (Chen et al., 2017). Recently, a rice 4CL4 belonging to 4-coumarate:coenzyme A ligases was reported to be involved in Al resistance (Liu et al., 2020).

Al-induced secretion of organic acids, including citrate, malate, and oxalate, has been well-documented as a very important mechanism by which plants resist the Al toxicity (Yang et al., 2019). Although transporters responsible for Al-induced citrate and malate secretion, respectively, have been characterized in a variety of plant species, genes encoding oxalate transporter remain unclear (Yang et al., 2019). Accumulating evidence suggests that oxalic acid has an important role in plant responses to both biotic (Molano-Flores, 2001; Jang et al., 2016) and abiotic stresses, including calcium regulation, ion homeostasis, metal stress, and other pathways (Palmieri et al., 2019). It has been reported that one of the AAE family members, AAE3 (acyl-activating enzyme3), is involved in oxalic acid metabolism–mediated plant growth and development and in resistance to biotic and abiotic stresses. For example, Foster et al. (2012) identified an *AAE3* gene encoding an oxalyl-CoA synthase in *Arabidopsis* and found that it participated in seed development and fungal pathogen defense by catalyzing CoA-dependent oxalate metabolism (Foster et al., 2012). Lou et al. (2016a) found that rice bean (*Vigna umbellata*) *VuAAE3* is involved in oxalate degradation and Al tolerance. Recently, Xian et al. (2020) also found that wild soybean *GsAAE3* similarly influences its tolerance to Cd and Al stress by catalyzing the oxalate metabolism. Therefore, it appears that AAE family proteins might have important roles in Al stress responses by regulating metabolic pathways.

In this study, we identified 53 *AAE* genes from tomato genome and found 9 differentially expressed *AAE* genes, including *SlAAE3-1* and *SlAAE3-2*, under Al stress. The expression pattern analysis of *SlAAE3-1* suggested that its expression is specific to Al stress. Therefore, our results contribute not only to enrich the molecular mechanism of Al stress response in tomato but also to provide a theoretical basis for improving tomato Al tolerance through genetic improvement and molecular breeding techniques.

## Materials and Methods

### Plant Materials and Grown Conditions

Tomato (*Solanum lycopersicum*) cultivar Ailsa Craig (AC) was used in this study (Horticulture Research International, Warwick, United Kingdom). The seeds were sterilized with 10% NaClO (v/v) for 15 min, washed thoroughly with sterile water, and soaking in sterilized water overnight. After that, the seeds were sown on Petri dishes containing 1/5 strength Hoagland nutrient solution (pH 5.5). The nutrient solution consisted of KNO_3_ (1.0 mM), Ca(NO_3_)_2_ (1.0 mM), MgSO_4_ (0.4 mM), and (NH_4_)H_2_PO_4_ (0.2 mM) and the micronutrients NaFeEDTA (20 μM), H_3_BO_3_ (3.0 μM), MnCl_2_ (0.5 μM), CuSO_4_ (0.2 μM), ZnSO_4_ (0.4 μM), and (NH_4_)_6_Mo_7_O_24_ (1 μM), with 0.8% agar (Sigma-Aldrich, Saint Louis, United States). Perti dishes were kept in the dark at 4°C for 2 days and then germinated in a plant growth room with a daytime 16 h/24°C and 8 h/22°C night regime. Germinated seedlings with uniform primary root length (4 cm) were transferred to 1/5 Hoagland nutrient solution (pH 5.5) with (NH_4_)H_2_PO_4_ concentration of 10 μM.

### Identification of Acyl-Activating Enzyme Superfamily in Tomato

The Hidden Markov Model (HMM) file corresponding to the AMP-bind domain (PF00501) was downloaded from the Pfam protein family database^1^ (El-Gebali et al., 2019). HMMER 3.2 was used to search against the *AAE* superfamily genes from the annotated tomato genome obtained from Phytozome version 12.1^2^ (Finn et al., 2011; Goodstein et al., 2012). All candidate genes that may contain AMP-binding domain based on HMMER results were further examined by confirming the existence of the AMP-binding core sequence using the PFAM and the SMART program^3^ (Letunic and Bork, 2018). The length of aa sequences, protein molecular weights (MWs), and isoelectric point of identified tomato AAE superfamily proteins were obtained by using tools from the ExPasy website.^4^

### Phylogenetic Analysis of Acyl-Activating Enzyme Supfamily Members

The sequences of 53 identified tomato AAEs and *Arabidopsis* all 60 AAE sequences according to two studies previously reported (Shockey et al., 2002, 2003; De Azevedo Souza et al., 2008) were used to create multiple protein sequence alignments using ClustalW in MEGA 7.0^5^ (Kumar et al., 2016) with default parameters. The alignment results were used to construct a phylogenetic tree using the neighbor-joining method with 1,000 bootstrap replicates. The phylogenetic tree was displayed using the R package ggtree (Yu et al., 2017).

### Gene Structure and Conserved Motif Analysis

The exon-intron distribution of each tomato *AAE* superfamily genes (*SlAAEs*) was analyzed by comparing predicted coding sequences with their corresponding genomic sequences using TBtools program (Chen et al., 2020). Conserved motifs of tomato AAE protein sequences were investigated using the online software MEME5.0.4^6^ (Bailey et al., 2009) with the following motif parameters: number of repetitions (any), maximum number of motif (20), and the optimum width of each motif (between 6 and 100 residues).

### Chromosomal Distribution and Gene Duplication Analysis

All *SlAAEs* were mapped to 12 tomato chromosomes based on physical location information from the database of tomato genome using TBtools (Chen et al., 2020). Multiple Collinearity Scan Toolkit, *McScanX* (Wang et al., 2012) with the default parameters was used to analyze the tandem repeats and segmental duplication events of *SlAAEs* superfamily in the tomato genome and synteny of *AAE* superfamily genes between tomato and *Arabidopsis*.

### Expression Analysis of Aluminum-Responsive *SlAAE* Genes

To investigate Al-responsive *SlAAEs*, the analysis of RNA-seq data (Jin et al., 2020) and qRT-PCR were performed. For qRT-PCR, seedlings were subjected to the modified 1/5 Hoagland nutrient solution (pH 5.0; 10 μM NH_4_H_2_PO_4_) containing 5 μM Al or 5 μM of CdCl_2_, or LaCl_3_, or 3 μM of CuCl_2_ for 6 h. RNA samples were extracted from both root tips (1 cm in length) after treatment. One microgram of DNA-free RNA was transcribed into first strand cDNA by PrimeScript RT Master Mix (TaKaRa). The qRT-PCR was carried out with the Roche LightCyler 480 instrument using SYBR Green chemistry (Toyobo, Osaka, Japan). The reaction conditions were 40 cycles at 95°C for 15 s, 60°C for 10 s, and 72°C for 15 s. The primer sequences used in this study are listed in Supplementary Table 1. Expression data of target genes were normalized with tomato *GAPDH* (Wang et al., 2016) by the ΔΔCt method. Each reaction was performed with three repeats from different biological samples.

### Promoter Analysis

The promoter data were obtained from Phytozome version 12.1 (see text footnote 2). The promoter analysis was conducted by searching 2.0 kb upstream sequences of the coding sequences against the PlantCARE database^7^ to identify their related *cis*-elements (Lescot et al., 2002). After sorting the *cis*-elements obtained from PlantCARE, the results were visualized and mapped to the AAE promoter using the TBtools software (Chen et al., 2020).

### Overexpression of *SlAAE3-1* in Tobacco and Aluminum Tolerance Evaluation

The open read frame of *SlAAE3-1* was amplified by PCR using gene-specific primer pair T***GGTACC***ATGGAGAGTATGACGCTC and CCG***GGATCC***CTACGCTCCAAATTTAGG, cloned into pCAMBIA1300 vector driven by Cauliflower mosaic virus 35S promoter and transformed into *Agrobacterium tumefaciens* (strain GV1301). Tobacco plants were transformed as described by Horsch et al. (1985). Transgenic lines carrying *SlAAE3-1* were selected using PCR with the primers described above. For evaluating Al tolerance of *SlAAE3-1*-overexpressing lines, seeds from T2 homozygous and wild-type lines were first sterilized, soaked, and germinated as described above. When the length of the primary root had reached about 4 cm, the seedlings were transferred to the modified 1/5 Hoagland nutrient solution (pH 5.0; 10 μM NH_4_H_2_PO_4_) containing 5 μM Al for 4 days. The solution was renewed every 2 days. The Al sensitivity was evaluated by relative root elongation expressed as (root elongation with Al treatment/root elongation without Al) × 100. After the treatment, root apex was stained with propidium iodide (PI) solution (5 μg/ml) for 1 min, washed with deionized water for 30 s, then observed, and captured using confocal laser scanning microscopy (Zeiss LSM710, Jena, Germany).

### Statistical Analysis

The Student’s *t*-test was performed in Microsoft Excel (version 2016, Microsoft Corp., Redmond, WA, United States). Data are given as means ± standard deviation (SD) of three independent biological replicates. A *p*-value < 0.05 was considered to be statistically significant.

## Results

### Identification of the Acyl-Activating Enzymes Superfamily in Tomato

We identified 53 members of AAEs superfamily in *S. lycopersicum* by searching the AAE consensus motif (PF00501) equal to PROSITE PS00455 (Shockey et al., 2003) following a previously described analysis pipeline (Jin et al., 2020). Gene characteristics, including the length of the coding sequence, the length of the protein sequence, the protein MW, isoelectric point (p*I*), and protein sequence, were analyzed (Supplementary Table 2). Among the 53 SlAAE proteins, Solyc07g043650 was identified to be the smallest protein with 455 aa, whereas the largest one was Solyc01g006640 (2,320 aa). The MW of SlAAE proteins ranged from 50.4 to 256.8 kDa, and the pI varied from 5.13 (Solyc08g076300) to 9.43 (Solyc03g005090).

### Phylogenetic Analysis and Classification of *SlAAE* Genes

**FIGURE 1:**
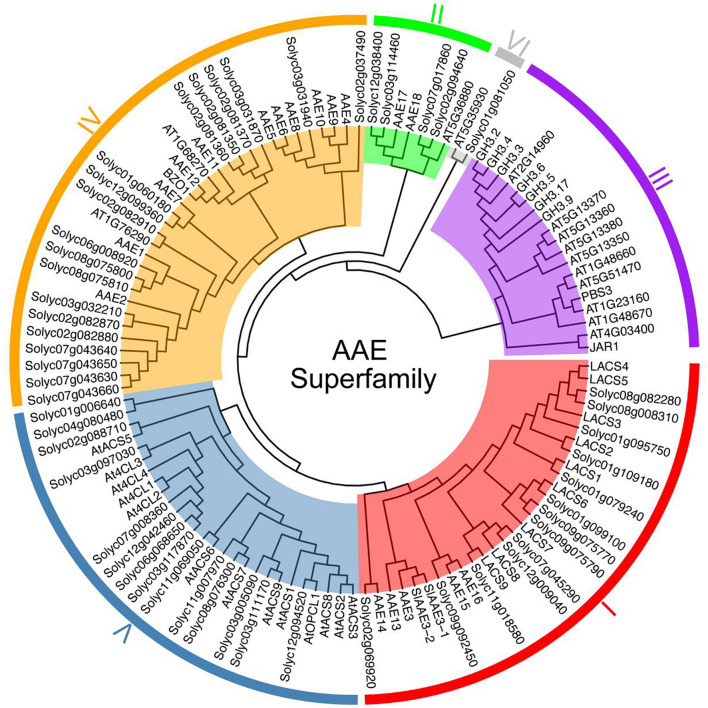
The phylogenetic analysis of tomato (*S. lycopersicum*) acyl-activating enzymes (AAEs) (SlAAEs). The phylogenetic analysis of AAEs from tomato and *Arabidopsis* using the complete protein sequences. The neighbor-joining (NJ) tree was constructed using the MEGA 7.0 software with the pairwise deletion option, and 1,000 bootstrap replicates were used to assess tree reliability. AAEs from tomato and *Arabidopsis* fell in six separate subfamilies as I–VI.

To probe the phylogenetic relationships among these 53 SlAAEs, we constructed an unrooted phylogenetic tree for SlAAEs together with 60 AtAAEs retrieved from previously published data (Shockey et al., 2002, 2003; De Azevedo Souza et al., 2008) by using neighbor-joining method. In consistent with previous result (Shockey et al., 2003), AAE superfamily could be separated into six distinct subfamilies (Figure 1). Among the 53 SlAAE proteins, 15 belong to clade I, 4 to clade II, 19 to clade IV (the largest clade), 14 to clade V, and 1 to clade VI (the smallest clade). Notably, clade III, a special subfamily, contains only 19 AtAAEs (Figure 1).

**FIGURE 2:**
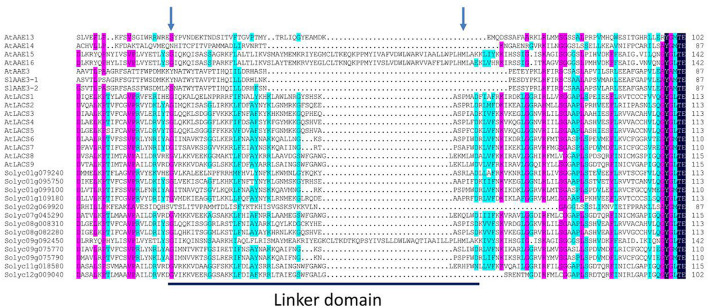
Multiple sequence alignment and linker domain structure of clade I proteins. The sequences of all acyl-activating enzyme (AAE) subfamily I proteins were aligned by DNAMAN software, and the sequence alignment and linker domain annotation were performed according to the reported sequence alignment results of *Arabidopsis* (Shockey et al., 2002). The blue arrows indicate the left and right boundaries of the linker domains, and the amino acid sequences in the horizontal line indicate the linker domains.

According to the feature of known long-chain acyl-CoA synthetase (LACS) proteins (Iijima et al., 1996; Fulda et al., 1997), 11 SlAAE members from clade I contain the eukaryote-type linker domain, a motif of between 30 and 70 aa residues (Figure 2), and may be active against long-chain fatty acids. However, similar to previously characterized AtLACSs (Shockey et al., 2002), the other four members (i.e., SlAAE3-1, SlAAE3-2, Solyc02g069920, and Solyc09g092450) probably do not produce the activity of the LACS enzyme even though they showed highly sequence similarity to 11 members. So far, the biological functions of 13 AAEs from clade I remain unknown except Solyc01g079240 (SlLACS1) and Solyc01g109180 (SlLACS2), both of which are reported to be involved in wound-induced suberization of tomato fruit (Han et al., 2018).

Six members of clade II could be further separated into two subgroups. Solyc02g094640 and Solyc07g017860 from clade II share 78% sequence similarity with AT5G63880 that was reported functioning as acetyl-CoA synthetase involved in lipid synthesis in seeds (Ke et al., 2000). The results of multiple sequence alignment among Solyc03g114460, Solyc12g038400, AAE17, and AAE18 revealed 69% sequence identity. However, all proteins from clade II share only 51% sequence similarity on average. These results suggest that these two subgroups might have distinct functions and require further biochemical assays to elucidate their functions.

Clade III consisted of 19 *Arabidopsis* AAE family members, termed adenylases, which are considered to participate in multiple important plant hormones (e.g., JA, IAA, and SA) signaling pathways through ATP-dependent adenylation of these hormones (Staswick et al., 2002; Shockey et al., 2003). Notably, no tomato AAEs were included in this clade. Among 19 tomato *AAE* genes in clade IV, only *Solyc07g043630* (*SlAACS1*) has been reported to be involved in biosynthesis of acylsugars in tomato trichomes (Fan et al., 2020), while the function of the remaining genes has not been characterized yet.

Clade V contains 13 putative *Arabidopsis 4-coumarate CoA ligases* (*At4Cls*) (Shockey et al., 2003) and 14 putative tomato *4CLs*. The 4CLs play a vital role in enhancing the mechanical support of plants and protecting plants from biotic and abiotic stresses depended on biosynthesis of lignins, flavonoids, and other compounds (Lavhale et al., 2018). For example, *4CL* gene involving in biosynthesis of lignin is upregulated in tomato (*S. lycopersicum*) upon *Alternaria solani* inoculation (Shinde et al., 2017). In rice, Al repressed the expression of *4CL4*, resulting in less lignin accumulation and more 4-coumaric acid and ferulic acid accumulation (Liu et al., 2020). In *Arabidopsis*, *At4CL1/2* required for biosynthesis of lignin was upregulated, while *At4CL3* involved in flavonoid was downregulated by wounding (Lee and Douglas, 1996; Ehlting et al., 1999; Soltani et al., 2006).

### Gene Structure and Protein Motif Analysis of *SlAAE* Genes

**FIGURE 3:**
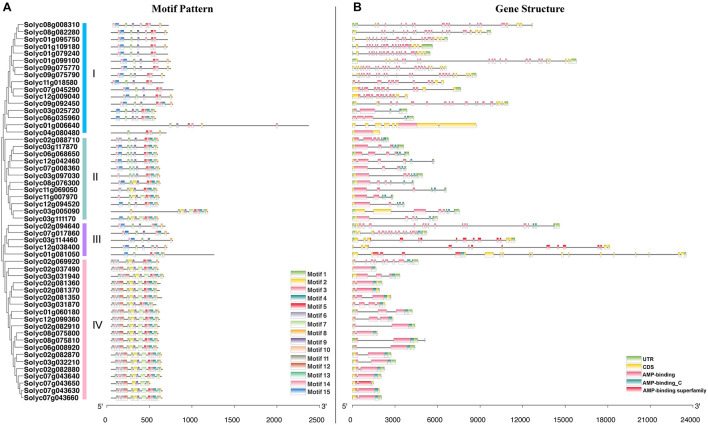
Phylogenetic relationships, gene structure, and architecture of conserved protein motifs in *AAE* genes from tomato. **(A)** The motif composition of tomato acyl-activating enzyme (AAE) proteins in accordance to the phylogenetic relationship. Fifteen motifs are shown in different colored boxes. The length of motifs in each AAE protein was displayed proportionally. **(B)** The exon-intron structure of *SlAAE* genes. Each exon-intron structure analysis of tomato *AAE* genes was completed using the online tool GSDS. The length of gene can be estimated using the scale at the bottom.

During the evolution of multigene families, diversification of gene structure may facilitate evolutionary co-option of genes for new functions to adapt to changing environments (Shang et al., 2013; Hu et al., 2015). To better understand the structural diversity of *SlAAE* genes, the conserved motifs and exon-intron organizations were analyzed according to the phylogenetic relationships (Figure 3).

**FIGURE 4:**
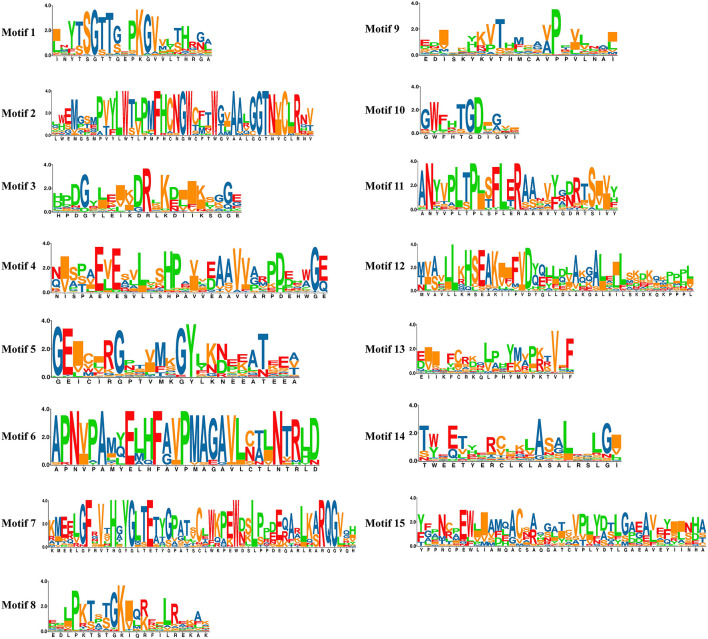
The sequence information of each conserved motif (1–15). Conserved motifs of acyl-activating enzyme (AAE) superfamily members were found using the MEME online tool with parameters: number of repetitions (any), maximum number of motif (20), and the optimum width of each motif (between 6 and 100 residues).

The potential conserved motifs of all SlAAE proteins were presented according to the MEME motif analysis as described (Xie et al., 2018). As a result, 15 different motifs were identified from all SlAAEs, which were successively named as motifs 1–15 (Figure 3A). The sequence information of each motif is displayed in Figure 4. Among these motifs, motifs 3, 5, and 9 belong to the AMP-binding domain, which are widely distributed on all SlAAEs. As expected, SlAAEs members within the same cluster in the phylogenetic tree commonly share a similar motif compositions (Figure 3A), indicating that they might have functional similarities.

As shown in Figure 3B and Supplementary Table 2, *SlAAEs* genes contain 0–22 introns. For instance, while *Solyc02g037490* and *Solyc04g080480* have no intron, *Solyc01g099100*, *Solyc09g075770*, and *Solyc09g075790* have 22 introns. Others have at least one intron, but genes with 8, 12, 14, 15, 20, and 21 introns were not observed. Most of *SlAAE* members in the same group have a similar gene structure. For example, most of group IV members contained only a single intron. These results suggest that SlAAEs possessing similar gene structures and motifs were clustered in the same group and might have evolved similar functions in tomato.

### Chromosomal Localization of *SlAAE* Genes

**FIGURE 5:**
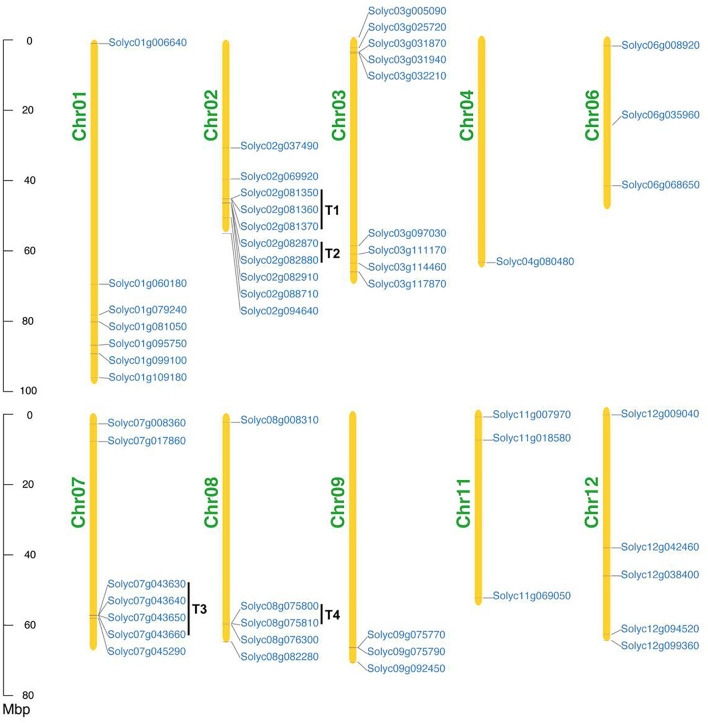
Chromosomal locations of *SlAAE* genes. In total, 53 *SlAAE* genes are distributed on 10 of 12 tomato chromosomes. Mbp indicates the scale. T, tandem duplication; Chr, chromosome.

To probe the chromosomal distribution of all *SlAAE* genes, all members were mapped to tomato chromosomes based on physical location information derived from the database of tomato genome. The result showed that all of the 53 *SlAAEs* could be mapped onto 10 out of 12 tomato chromosomes (except Chr05 and Chr10) in an increasing order from short-arm to long-arm telomere (Figure 5), and most of *SlAAE* genes were distributed in the chromosome ends. In addition, bias change in gene number was inspected. Among 10 chromosomes, Chr02 contained largest number of *AAE* genes, while Chr04 had least.

According to a previous study (Holub, 2001), 200 kb of a chromosomal area containing two or more genes is defined as a tandem duplication event. As shown in Figure 5, four gene pairs present as tandem duplication (T) observed on three chromosomes, i.e., T1 (*Solyc02g081350*, *Solyc02g081360*, and *Solyc02g081370*) and T2 (*Solyc02g082870* and *Solyc02g082880*) on Chr02, T3 (*Solyc07g043630*, *Solyc07g043640*, *Solyc07g043650*, and *Solyc07g043660*) on Chr07, and T4 (*Solyc08g075800* and *Solyc08g075810*) on Chr08. Therefore, in-tandem AAE duplicates comprise 21% of the whole tomato AAE superfamily.

### Syntenic Analysis of *AAE* Genes in Tomato Genome

**FIGURE 6:**
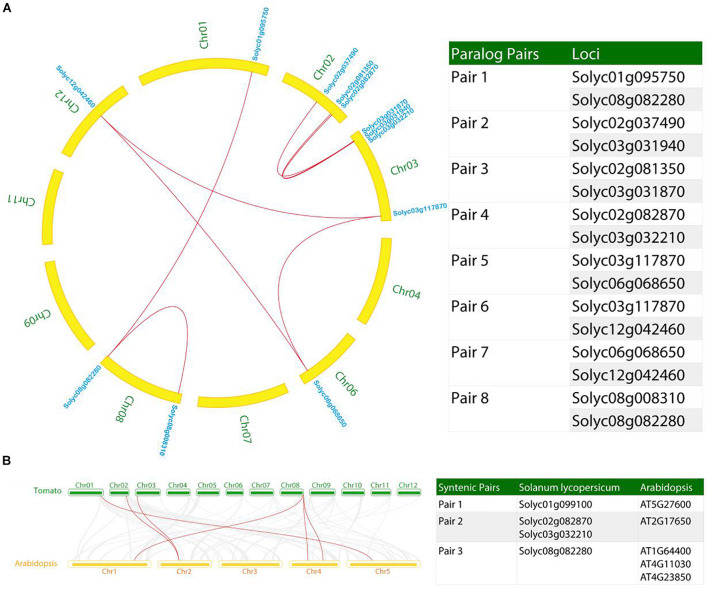
Syntenic analysis of *AAE* genes in the tomato genome. **(A)** Identification of paralog pairs in syntenic blocks within the tomato genome. Ten paralog pairs were identified. **(B)** Synteny analysis of *AAE* genes between tomato and *Arabidopsis*. Three syntenic pairs were found in this analysis. Gray lines in the background indicate the collinear blocks within tomato and *Arabidopsis*, while the red lines highlight the syntenic *AAE* gene pairs.

Besides the tandem duplication events, we also investigated the segmental duplication in the tomato genome relating to the recurring polyploidization events, which generated gene duplicates that have usually been retained in extant tomato genome (Wang and Paterson, 2011). In this study, Eight pairs (named pair 1–8) of syntenic AAE paralogs were observed within the tomato genome (Figure 6A). According to the phylogenetic tree (Figure 1), the tomato paralogs belong to clade I (syntenic pairs 1 and 8), clade IV (syntenic pairs 2, 3, and 4), as well as clade V (syntenic pairs 5, 6, and 7), thus allowing us to propose the conserved functions between the syntenic pairs.

Furthermore, we constructed a comparative syntenic map between tomato and *Arabidopsis* (Figure 6B). Three syntenic pairs comprising four *SlAAE* genes were identified in syntenic blocks. We also found that duplicated *AAE* genes exhibited conserved synteny with *Arabidopsis* genes. For example, *Solyc02g082870* is microsyntenic to *Solyc03g032210* (paralog pair 4), both of which are syntenic to *AT2G17650* (ortholog pair 2, Figure 6B). *Solyc08g082280* is microsyntenic to *Solyc01g095750* (paralog pair 1) and syntenic to three *Arabidopsis* genes (ortholog pair 3). Based on these information, we could have inferred the functions of these tomato genes based on the functions of *Arabidopsis* genes, though the functions of these genes in *Arabidopsis* are yet to be investigated. Nevertheless, the biological functions of these ortholog genes could have been conservatively evolved since the last common ancestor of tomato and *Arabidopsis*, which is estimated to have existed approximately 150 million years ago (Ku et al., 2000).

### Expression Profiles of *SlAAEs* Under Aluminum Stress

**FIGURE 7:**
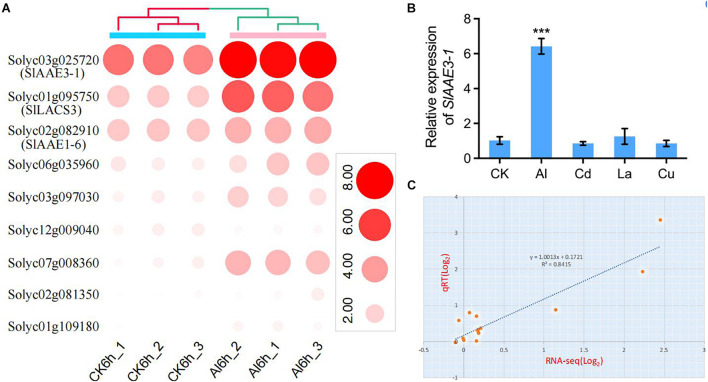
Expression profiles of *SlAAEs* under Al stress. **(A)** Heatmap of nine differentially expressed *SlAAE* genes during Al stress. **(B)** Expression specificity of *SlAAE3-1* gene. Seedlings were subjected to 1/5 Hoagland solution containing 5 μM AlCl_3_, or 5 μM CdCl_2_, 5 μM LaCl_3_, or 3 μM CuCl_2_ for 6 h. Data are the means ± SD (*n* = 3); the asterisk indicates significant differences between control and treatment at *P <* 0.001 using one-way ANOVA. **(C)** Correlation of gene expression levels between the RNA-seq data and quantitative reverse-transcriptase (qRT-PCR) analysis. Fourteen *SlAAEs* potentially responding to Al were selected and subjected to the qRT-PCR analysis using the same RNA as for RNA-seq. Both *x*- and *y*-axes are shown in Log_2_ scale.

We have previously demonstrated that rice bean (*V. umbellata*) *VuAAE3*, a gene showing a high sequence identity to *SlAAE3-1* (*Solyc03g025720*) and *SlAAE3-2* (*Solyc06g035960*), was involved in Al tolerance (Lou et al., 2016a). This prompted us to investigate the potential function of *SlAAE* genes in Al stress response after a systemic analysis of the *SlAAE* gene family. On the basis of tomato root tip Al stress-responsive expressed genes identified from the results of RNA-seq (SRP227103) (Jin et al., 2020), we found that 50 out of 53 *SlAAE* genes could be detected by RNA-seq in the tomato root apex (Supplementary Table 3). However, six *AAE* genes showed FPKM value lower than six and were hardly expressed either without or with Al stress. In addition, the expression of 35 *AAE* genes was not induced by Al stress in tomato root apexes (Supplementary Table 3). Finally, only 9 out of 53 *SlAAE*s were identified to be differentially regulated, 8 were upregulated, and 1 were downregulated by 5 μM of Al (Supplementary Table 4 and Figure 7A). Notably, *Solyc03g025720* (*SlAAE3-1*) was highly expressed and greatly induced by Al compared with others. We then examined the specificity of *SlAAE3-1* expression by exposing tomato seedlings to various metals, including Al, Cd, La, and Cu. The expression of *SlAAE3-1* was greatly induced by Al but not by other metals (Figure 7B). To verify the reliability of the RNA-seq data, 14 *SlAAE* genes were selected for the qRT-PCR analysis. As shown in Figure 7C, all 14 *SlAAE* genes displayed similar expression patterns to that obtained using RNA-seq. A good correlation (*R*^2^ = 0.8415) was observed for their expression in plot qRT-PCR results against that of RNA-seq, indicating that the RNA-seq data accurately reflected the transcriptional changes induced by Al stress.

### Identification of Stress-Responsive *cis-*Acting Elements of *SlAAEs*

**FIGURE 8:**
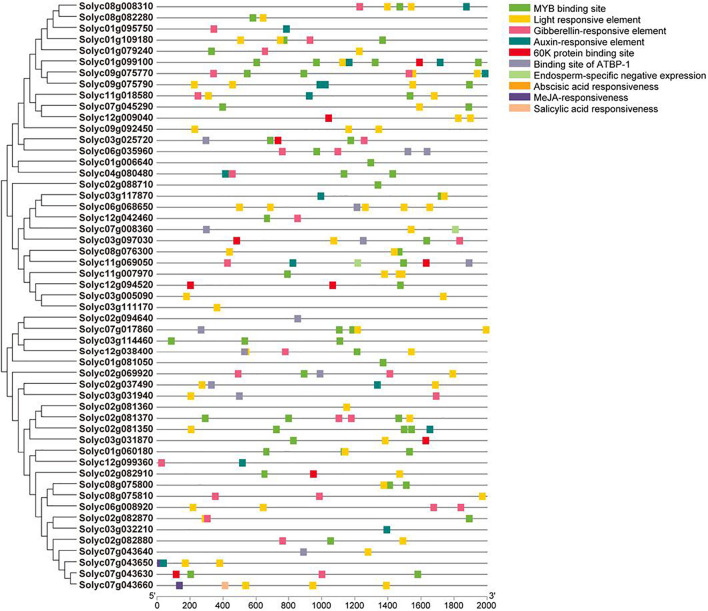
Phylogenetic and *cis*-element analysis of *SlAAE* family promoters. A total of 53 of promoter sequences from tomato genome were scanned using PlantCARE. Sorted *cis*-elements were then mapped on promoters of the corresponding *AAEs* and visualized using TBtools. Colored rectangles represent different *cis*-element.

To explore the *cis*-acting elements probably implicated in the expression regulation of *SlAAE*s, 2.0 kb sequences upstream of the start codon of *SlAAEs* were analyzed using the PlantCARE database (Lescot et al., 2002). The results showed that most of the predicted *cis*-acting elements were associated with phytohormone responses. In addition, there were MYB-binding site, light-responsive element, 60K protein site, ATBP-1 binding site, and endosperm-specific negative expression. However, we did not find Al-responsive element predicted from the PlantCARE (Figure 8). Sensitive to proton rhizotoxicity 1 (STOP1) is a C2H2-type zinc finger transcription factor that regulates expression of many downstream genes involved in Al tolerance by binding to STOP1 *cis*-acting elements GGN(T/g/a/C)V(C/A/g)S(C/G) present in their promoters (Tsutsui et al., 2011; Liu et al., 2016). Therefore, we analyzed the 2 kb length promoter sequence of *SlAAE3-1* and identified 28 *cis*-acting elements that have potentials to interact with STOP1 (Table 1), suggesting that the STOP1 regulatory module may also be present in tomato.

**TABLE 1:** The number and position of sensitive to proton rhizotoxicity 1 (STOP1) *cis*-acting elements of *SlAAE3-1* promoter.

**Motif sequence**	**Position of STOP1 *cis*-element**	**No. of STOP1 *cis*-element**
GGGAG	–2, –420, –463, –553	4
GGGGC	–320	1
GGGGG	–461	1
GGACC	–1,038	1
GGACG	–1,480	1
GGAAG	–521, –606, –1,836	3
GGAGC	–552	1
GGAGG	–123, –421, –1477	3
GGCCC	–181	1
GGCAC	–304, –427, –472	3
GGCAG	–1,398	1
GGTCC	–1,038	1
GGTCG	–296	1
GGTAC	–1,000	1
GGTAG	–1,535	1
GGTGC	–1,145, –1,363	2
GGTGG	–424, –1,010	2

### Effect of *SlAAE3-1* Overexpression in Tobacco on the Tolerance to Aluminum

**FIGURE 9:**
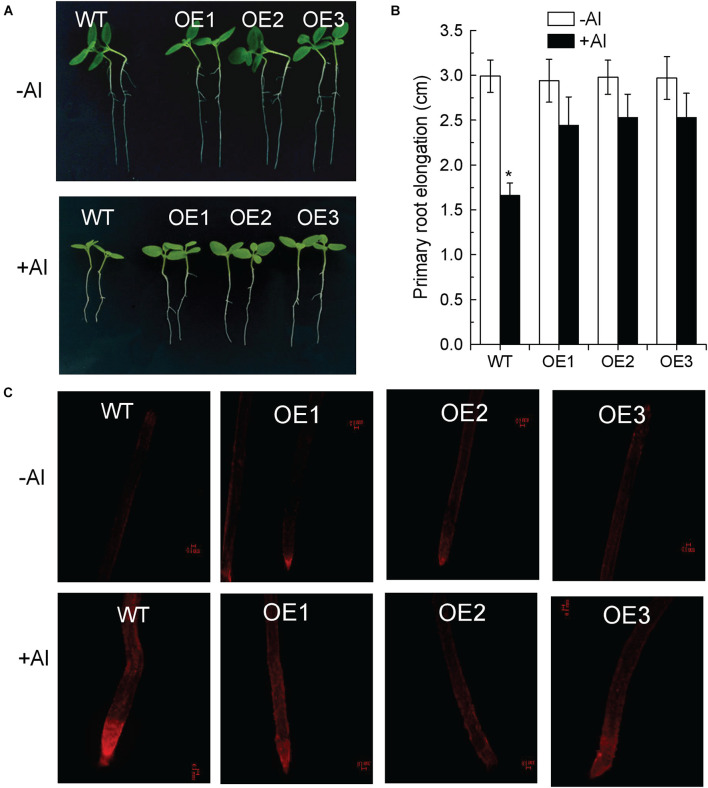
Overexpression of SlAAE3-1 in tobacco improved Al tolerance. **(A)** Al tolerance phenotype of *SlAAE3-1* overexpressing tobacco lines. **(B)** Primary root elongation. Data are the means ± standard deviation (SD) (*n* = 10); the asterisk indicates significant differences between control and treatment at *P <* 0.001 using one-way ANOVA. **(C)** Propidium iodide staining, fluorescence signals were analyzed using confocal microscopy, bars = 100 μm.

To confirm that the identified differentially expressed *SlAAE* genes are exactly involved in tolerance to stresses, we developed transgenic tobacco lines overexpressing *SlAAE3-1* that is most significantly induced by Al in the tomato root apex. Three independent *SlAAE3-1* overexpressing tobacco lines (i.e., OE1, OE2, and OE3) were selected for examining their tolerance to Al stress. Under normal condition, the wild-type (WT) and transgenic lines showed no difference in root elongation. However, in the presence of 5 μM of Al, the elongation of the primary root of transgenic lines was significantly greater than WT lines (Figures 9A,B). In addition, the PI staining was used to check cell damage. In the absence of Al, PI was hardly stained both in the WT roots and transgenic lines (Figure 9C). However, Al stress resulted in the red fluorescence signals to be more severely accumulated in the WT root apex than in the transgenic lines (Figure 9C), suggesting that transgenic tobacco lines were more tolerant to Al stress than WT plants. Therefore, *SlAAE3-1* plays roles in the tolerance to Al stress, consisting with its transcriptional regulation by Al.

## Discussion

In higher plants, AAEs, also called acyl adenylate-forming (Conti et al., 1996; Chang et al., 1997) or AMP-binding proteins (Fulda et al., 1997), are involved in numerous metabolic pathways, such as fatty acid β-oxidation (Schnurr et al., 2004), oxalate catabolism (Foster et al., 2016), and malonic acid degradation (Chen et al., 2011). In the current study, we systemically analyzed the AAE superfamily in tomato and identified a total of 53 members (Supplementary Table 2). We further divided tomato *AAE* superfamily into five distinct clades based on the phylogenetic analysis (Figure 1). *SlAAEs* and *AtAAEs* from clades I, II, IV, V, and VI showed that these genes were not only homologous but could be evolved from a common ancestor. However, *AAEs* from clade III indicated that this clade of *AtAAE* genes had a different ancestor with tomato (Figure 1). It is also interesting to note that clade III AAEs encompass 19 *Arabidopsis* plant hormone adenylases including JAR1. Substrate-dependent ATP-^32^P-PPi isotope exchange experiment demonstrated that JAR1 is specifically active on JA, while some members from this clade are active on auxin (Staswick et al., 2002). Here, we found that all 53 tomato *AAE* superfamily members had hardly been reported functionally, except *SlLACS1* (*Solyc01g079240*) and *SlLACS2* (*Solyc01g109180*) (Han et al., 2018). Therefore, the systematic analysis and identification of the tomato *AAE* superfamily in this study facilitate further studying on the biological function of the superfamily.

As one of the most important metabolisms in plants, phenylpropanoid metabolism provides precursors for more than 8,000 metabolites contributing to plant development and plant-environment interplay (Lavhale et al., 2018; Dong and Lin, 2021). The reaction catalyzed by 4-coumarate-CoA ligase (4CL) is the third step of the first three shared common steps of the general phenylpropanoid pathway, which is responsible for channelizing precursors for various phenylpropanoids (Fraser and Chapple, 2011; Lavhale et al., 2018; Dong and Lin, 2021). In plants, 4CL enzymes belong to AAE superfamily and catalyze the reaction that converts methoxy or hydroxycinnamic acid derivatives to corresponding CoA thioesters (Shockey et al., 2003; Lavhale et al., 2018). In addition, the 4CL enzymes play vital roles in plant physiology or in responses to biotic and abiotic stresses (Fritzemeier et al., 1987; Lee and Douglas, 1996; Sun et al., 2013; Abdollahi Mandoulakani et al., 2017; Blanco-Ulate et al., 2017). In rice, 4CL4-knockout mutants increase the Al tolerance by reducing the binding of Al to the cell walls caused by increased accumulation of 4-coumaric acid and ferulic acid that strengthens the cross-linking of the hemicellulose (Liu et al., 2020). In this study, we also identified 14 putative tomato 4CL and 4CL-like enzymes in clade V, but their biological functions have to be characterized in future. According to the RNA-seq data, we found that the expression level of 2 *4CL* genes, *Solyc03g097030* and *Solyc06g035960*, increased under Al stress for 6 h (Figure 7A). *4CLs* in dicots, such as tomato and *Arabidopsis*, could be grouped into two clusters, namely, type I and type II (Supplementary Figure 1). Type I is mainly involved in lignin biosynthesis, whereas type II cluster is involved in phenylpropanoid biosynthesis other than lignin (Gui et al., 2011; Lavhale et al., 2018). However, in monocot plants, such as rice, five *Os4CLs* were mainly categorized into type III except *Os4CL2* that belongs to type II (Gui et al., 2011; Sun et al., 2013). As shown in Supplementary Figure 1, *Solyc03g097030* belongs to type I, indicating that *Solyc03g097030* responds to Al stress possibly by regulating lignin biosynthesis. However, it remains possible that *Solyc03g097030* might be involved in other biosynthesis pathways, not just lignin biosynthesis. This proposition is supported by a recent report that although *Os4CL4* belongs to type III, it does participate in the regulation of lignin biosynthesis (Liu et al., 2020).

In general, the presence of multiple paralogs in multigene families may relate to the recurring polyploidization events of the angiosperm lineage, which generated gene duplicates that have often retained in extant plant genomes (Wang and Paterson, 2011). It has been shown that a genome-wide duplication event happened in tomato about 83–123 Myr (Sato et al., 2012). Over time, these gene duplicates may have culminated in sub- or neofunctionalization and, subsequently, acquired new functions that are occasionally retained, thus resulting in functional diversity and proliferation of genes derived from a common ancestor gene (Veitia, 2005). In this study, we revealed four tandem duplication segments (Figure 5), which may result in an intensification of gene expression. For example, maize with in-tandem MATE genes (three-copy allele) show a greater Al tolerance as enhanced overall expression of these genes (Maron et al., 2013). Orthologs and paralogs are two essentially different types of homologous genes that are associated with speciation or duplication (Koonin, 2005; Gabaldón and Koonin, 2013). In current study, eight pairs of syntenic AAE paralogs were found within the tomato genome (Figure 6A), and three ortholog pairs were identified in syntenic blocks between tomato and *Arabidopsis* (Figure 6B). According to the phylogenetic tree, we found that tomato paralog pair 4 belong to the clade IV (Figures 1, 6A), and this paralog pair showed synteny with *Arabidopsis AT2G17650* (ortholog pair 2 in Figure 6B), suggesting that these genes could share conserved functions. These results suggested that the analysis of synteny of genes contributes to inferring novel gene functions based on known genes.

Long-chain acyl-CoA synthetases represent a subgroup of AAE superfamily that activates free fatty acids to acyl-CoA and as such play vital roles in long-chain or very-long-chain fatty acids metabolism (Shockey et al., 2002; Shockey and Browse, 2011; Zhao et al., 2021). The loss of catalytic activity of LACS often causes pleiotropic phenotypes such as organ fusion (Weng et al., 2010), male sterility (Jessen et al., 2011), deficient cuticle (Ingram and Nawrath, 2017), delayed seed germination (Shockey et al., 2002), and plant ability to respond to various environmental stresses including drought (Bessire et al., 2007; Lü et al., 2009; Zhao et al., 2019), hypoxia (Licausi et al., 2011; Schmidt et al., 2018; Schmidt and van Dongen, 2019; Xie et al., 2020), and biotic stress (Bessire et al., 2007; Tang et al., 2007; Lü et al., 2009). It has been shown that eukaryotic-type *LACSs* usually contain the linker domain (Shockey et al., 2002). Combining the phylogenetic tree (Figure 1) and the result of multiple sequence alignment between clade I members (Supplementary Figure 1), We identified 11 tomato LACS members containing a linker domain and nine known AtLACS (1–9) (Shockey et al., 2002). Interestingly, although four tomato members (i.e., SlAAE3-1, SlAAE3-2, Solyc02g069920, and Solyc09g092450) and five *Arabidopsis* members (i.e., AtAAE3, AtAAE13/14/15/16) belong to clade I-like LACSs, these enzymes do not contain a linker domain. AAE3 has been reported to be involved in oxalate degradation (Foster et al., 2016); however, whether it is involved in other metabolic pathways remains unknown. The above analysis suggests that AAE3 may be involved in fatty acid metabolism in addition to long-chain (i.e., LCFAs; C16–C18) or very-long-chain fatty acids (i.e., VLCFAs; ≥C20), which preferentially activated by LACSs (Shockey et al., 2002; Lü et al., 2009; Shockey and Browse, 2011; Zhao et al., 2021). But, the function of AAE3 still needs further study.

We identified nine tomato *SlAAE* genes that rapidly responded to Al stress in the tomato root apex, among which *SlAAE3-1* was most abundantly expressed and dramatically upregulated (Figure 7 and Supplementary Table 4). Previous studies have shown that rice bean *VuAAE3* and wild soybean *GsAAE3* are implicated in Al tolerance by regulating oxalate acetylation (Lou et al., 2016a; Xian et al., 2020). Here, we demonstrated that tomato *SlAAE3-1* plays the same role with respect to Al tolerance (Figure 9). However, the role of other Al-responsive *SlAAE* genes in Al tolerance has to be investigated. There is considerable evidence that *AAE* genes play critical roles in the plant growth and development (Schnurr et al., 2004; Lü et al., 2009; Chen et al., 2011; Yang et al., 2017) and improving tolerance to abiotic stresses, including salinity (Zhou et al., 2020), drought (Bessire et al., 2007; Zhao et al., 2019), and metal stress (e.g., Cd and Al) (Lou et al., 2016a; Xian et al., 2020). Therefore, the functional roles of these *SlAAEs* in various stress responses could be inferred. In accordance with this supposition, many *cis-*acting elements related to biotic and abiotic stresses have been identified to be present in their promoters (Figure 8).

In summary, we provided the first integrated bioinformatic information of SlAAE superfamily in tomato including gene identification, structure, chromosomal location, duplication, and expression regulation by Al stress. A total of 53 *SlAAE* genes were identified, which is essential for the functional characterization of *SlAAE3* genes in tomato in future. Furthermore, the RNA-seq data and qRT-PCR analysis have identified nine Al-responsive *SlAAE* genes and characterized one of them, SlAAE3-1, to be implicated in Al tolerance, which pave the way for identifying novel genes involved in Al tolerance. In addition, the central role of SlAAE members in diverse metabolisms shed light on the importance of this family in responding to both biotic and abiotic stresses.

## Data Availability Statement

The original contributions presented in the study are included in the article/Supplementary Material, further inquiries can be directed to the corresponding authors.

## Author Contributions

JY and WC conceived the research. JJ, QH, and PL performed the experiments. HL provided the technical assistance. JJ and QH analyzed the data. JJ, WC, and JY wrote the manuscript. All authors contributed to the article and approved the submitted version.

## Conflict of Interest

The authors declare that the research was conducted in the absence of any commercial or financial relationships that could be construed as a potential conflict of interest.

## Publisher’s Note

All claims expressed in this article are solely those of the authors and do not necessarily represent those of their affiliated organizations, or those of the publisher, the editors and the reviewers. Any product that may be evaluated in this article, or claim that may be made by its manufacturer, is not guaranteed or endorsed by the publisher.

## Footnotes

1
http://pfam.xfam.org/

2
https://phytozome.jgi.doe.gov/pz/portal.html

3
http://smart.embl-heidelberg.de/smart/batch.pl

4
https://www.expasy.org/

5
https://www.megasoftware.net/

6
http://meme-suite.org/tools/meme

7
http://bioinformatics.psb.ugent.be/webtools/plantcare/html/

## References

[B1] Abdollahi Mandoulakani B. Eyvazpour E. Ghadimzadeh M. (2017). The effect of drought stress on the expression of key genes involved in the biosynthesis of phenylpropanoids and essential oil components in basil (*Ocimum basilicum* L.). *Phytochemistry* 139 1–7. 10.1016/j.phytochem.2017.03.006 28366608

[B2] Bailey T. L. Boden M. Buske F. A. Frith M. Grant C. E. Clementi L. (2009). MEME SUITE: tools for motif discovery and searching. *Nucleic Acids Res.* 37 202–208. 10.1093/nar/gkp335 19458158 PMC2703892

[B3] Bessire M. Chassot C. Jacquat A. C. Humphry M. Borel S. Petétot J. M. C. (2007). A permeable cuticle in *Arabidopsis* leads to a strong resistance to *Botrytis cinerea*. *EMBO J.* 26 2158–2168. 10.1038/sj.emboj.7601658 17396154 PMC1852784

[B4] Blanco-Ulate B. Hopfer H. Figueroa-Balderas R. Ye Z. Rivero R. M. Albacete A. (2017). Red blotch disease alters grape berry development and metabolism by interfering with the transcriptional and hormonal regulation of ripening. *J. Exp. Bot.* 68 1225–1238. 10.1093/jxb/erw506 28338755 PMC5444480

[B5] Chang K. H. Xiang H. Dunaway-Mariano D. (1997). Acyl-adenylate motif of the acyl-adenylate/thioester-forming enzyme superfamily: a site-directed mutagenesis study with the *pseudomonas* sp. strain CBS3 4-chlorobenzoate:coenzyme A ligase. *Biochemistry* 36 15650–15659. 10.1021/bi971262p 9398293

[B6] Chen C. J. Chen H. Zhang Y. Thomas H. R. Frank M. H. He Y. H. (2020). TBtools: an integrative toolkit developed for interactive analyses of big biological data. *Mol. Plant* 13 1194–1202. 10.1016/j.molp.2020.06.009 32585190

[B7] Chen G. Liu H. Wei Q. Zhao H. Liu J. Yu Y. (2017). The acyl-activating enzyme PhAAE13 is an alternative enzymatic source of precursors for anthocyanin biosynthesis in petunia flowers. *J. Exp. Bot.* 68 457–467. 10.1093/jxb/erw426 28204578 PMC5441920

[B8] Chen H. Kim H. U. Weng H. Browse J. (2011). Malonyl-CoA synthetase, encoded by ACYL ACTIVATING ENZYME13, is essential for growth and development of *Arabidopsis*. *Plant Cell* 23 2247–2262. 10.1105/tpc.111.086140 21642549 PMC3160029

[B9] Conti E. Franks N. P. Brick P. (1996). Crystal structure of firefly luciferase throws light on a superfamily of adenylate-forming enzymes. *Structure* 4 287–298. 10.1016/S0969-2126(96)00033-0 8805533

[B10] De Azevedo Souza C. Barbazuk B. Ralph S. G. Bohlmann J. Hamberger B. Douglas C. J. (2008). Genome-wide analysis of a land plant-specific acyl:coenzymeA synthetase (ACS) gene family in *Arabidopsis*, poplar, rice and *Physcomitrella*. *New Phytol.* 179 987–1003. 10.1111/j.1469-8137.2008.02534.x 18627494

[B11] Dong N. Q. Lin H. X. (2021). Contribution of phenylpropanoid metabolism to plant development and plant–environment interactions. *J. Integr. Plant Biol.* 63 180–209. 10.1111/jipb.13054 33325112

[B12] Ehlting J. Büttner D. Wang Q. Douglas C. J. Somssich I. E. Kombrink E. (1999). Three 4-coumarate:coenzyme A ligases in *Arabidopsis thaliana* represent two evolutionarily divergent classes in angiosperms. *Plant J.* 19 9–20. 10.1046/j.1365-313X.1999.00491.x 10417722

[B13] El-Gebali S. Mistry J. Bateman A. Eddy S. R. Luciani A. Potter S. C. (2019). The Pfam protein families database in 2019. *Nucleic Acids Res.* 47 427–432. 10.1093/nar/gky995 30357350 PMC6324024

[B14] Fan P. X. Wang P. P. Lou Y. R. Leong B. J. Moore B. M. Schenck C. A. (2020). Evolution of a plant gene cluster in Solanaceae and emergence of metabolic diversity. *eLife* 9:e56717. 10.7554/eLife.56717 32613943 PMC7386920

[B15] Finn R. D. Clements J. Eddy S. R. (2011). HMMER web server: interactive sequence similarity searching. *Nucleic Acids Res.* 39 29–37. 10.1093/nar/gkr367 21593126 PMC3125773

[B16] Foster J. Kim H. U. Nakata P. A. Browse J. (2012). A previously unknown oxalyl-CoA synthetase is important for oxalate catabolism in *Arabidopsis*. *Plant Cell* 24 1217–1229. 10.1105/tpc.112.096032 22447686 PMC3336115

[B17] Foster J. Luo B. Nakata P. A. (2016). An oxalyl-CoA dependent pathway of oxalate catabolism plays a role in regulating calcium oxalate crystal accumulation and defending against oxalate-secreting phytopathogens in *Medicago truncatula*. *PLoS One* 11:e0149850. 10.1371/journal.pone.0149850 26900946 PMC4763187

[B18] Fraser C. M. Chapple C. (2011). The phenylpropanoid pathway in *Arabidopsis*. *Arab. Book* 9:e0152. 10.1199/tab.0152 22303276 PMC3268504

[B19] Fritzemeier K. H. Cretin C. Kombrink E. Rohwer F. Taylor J. Scheel D. (1987). Transient induction of phenylalanine ammonia-lyase and 4-coumarate: CoA ligase mRNAs in potato leaves infected with virulent or avirulent races of *Phytophthora infestans*. *Plant Physiol.* 85 34–41. 10.1104/pp.85.1.34 16665678 PMC1054198

[B20] Fulda M. Heinz E. Wolter F. P. (1997). *Brassica napus* cDNAs encoding fatty acyl-CoA synthetase. *Plant Mol. Biol.* 33:12. 10.1023/A:1005780529307 9106514

[B21] Gabaldón T. Koonin E. V. (2013). Functional and evolutionary implications of gene orthology. *Nat. Rev. Genet.* 14 360–366. 10.1038/nrg3456 23552219 PMC5877793

[B22] Goodstein D. M. Shu S. Howson R. Neupane R. Hayes R. D. Fazo J. (2012). Phytozome: a comparative platform for green plant genomics. *Nucleic Acids Res.* 40 1178–1186. 10.1093/nar/gkr944 22110026 PMC3245001

[B23] Gui J. Shen J. Li L. (2011). Functional characterization of evolutionarily divergent 4-coumarate:coenzyme A ligases in rice. *Plant Physiol.* 157 574–586. 10.1104/pp.111.178301 21807887 PMC3192572

[B24] Han X. Y. Mao L. Lu W. J. Tao X. Y. Wei X. P. Luo Z. S. (2018). Abscisic acid induces differential expression of genes involved in wound-induced suberization in postharvest tomato fruit. *J. Integr. Agric.* 17 2670–2682. 10.1016/s2095-3119(18)62142-2

[B25] Holub E. B. (2001). The arms race is ancient history in *Arabidopsis*, the wildflower. *Nat. Rev. Genet.* 2 516–527. 10.1038/35080508 11433358

[B26] Horsch R. B. Fry J. E. Hoffmann N. L. Eichholtz D. Rogers S. G. Fraley R. T. (1985). A simple and general method for transferring genes into plants. *Science* 227, 1229–1231. 10.1126/science.227.4691.1229 17757866

[B27] Hu W. Wei Y. X. Xia Z. Q. Yan Y. Hou X. W. Zou M. L. (2015). Genome-wide identification and expression analysis of the NAC transcription factor family in cassava. *PLoS One* 10:e0136993. 10.1371/journal.pone.0136993 26317631 PMC4552662

[B28] Iijima H. Fujino T. Minekura H. Suzuki H. Kang M. J. Yamamoto T. (1996). Biochemical studies of two rat acyl-CoA synthetases, ACS1 and ACS2. *Eur. J. Biochem.* 242 186–190. 10.1111/j.1432-1033.1996.0186r.x 8973631

[B29] Ingram G. Nawrath C. (2017). The roles of the cuticle in plant development: organ adhesions and beyond. *J. Exp. Bot.* 68 5307–5321. 10.1093/jxb/erx313 28992283

[B30] Jang J. Y. Choi Y. H. Shin T. S. Kim T. H. Shin K. S. Park H. W. (2016). Biological control of *Meloidogyne incognita* by *Aspergillus niger* F22 producing oxalic acid. *PLoS One* 11:e0156230. 10.1371/journal.pone.0156230 27258452 PMC4892604

[B31] Jessen D. Olbrich A. Knüfer J. Krüger A. Hoppert M. Polle A. (2011). Combined activity of LACS1 and LACS4 is required for proper pollen coat formation in *Arabidopsis*. *Plant J.* 68 715–726. 10.1111/j.1365-313x.2011.04722.x 21790813

[B32] Jin J. F. Wang Z. Q. He Q. Y. Wang J. Y. Li P. F. Xu J. M. (2020). Genome-wide identification and expression analysis of the NAC transcription factor family in tomato (*Solanum lycopersicum*) during aluminum stress. *BMC Genomics* 21:288. 10.1186/s12864-020-6689-7 32264854 PMC7140551

[B33] Ke J. S. Behal R. H. Back S. L. Nikolau B. J. Wurtele E. S. Oliver D. J. (2000). The role of pyruvate dehydrogenase and acetyl-coenzyme A synthetase in fatty acid synthesis in developing *Arabidopsis* seeds. *Plant Physiol.* 123 497–508. 10.1104/pp.123.2.497 10859180 PMC59018

[B34] Kienow L. Schneider K. Bartsch M. Stuible H. P. Weng H. Miersch O. (2008). Jasmonates meet fatty acids: functional analysis of a new acyl-coenzyme A synthetase family from *Arabidopsis thaliana*. *J. Exp. Bot.* 59 403–419. 10.1093/jxb/erm325 18267944

[B35] Kochian L. V. (1995). Cellular mechanisms of aluminum toxicity and resistance in plants. *Annu. Rev. Plant Physiol. Plant Mol. Biol.* 46 237–260. 10.1146/annurev.pp.46.060195.001321

[B36] Kochian L. V. Hoekenga O. A. Piñeros M. A. (2004). How do crop plants tolerate acid soils? Mechanisms of aluminum tolerance and phosphorous efficiency. *Annu. Rev. Plant Biol.* 55 459–493. 10.1146/annurev.arplant.55.031903.141655 15377228

[B37] Kochian L. V. Piñeros M. A. Liu J. P. Magalhaes J. V. (2015). Plant adaptation to acid soils: the molecular basis for crop aluminum resistance. *Annu. Rev. Plant Biol.* 66 571–598. 10.1146/annurev-arplant-043014-114822 25621514

[B38] Koo A. J. K. Chung H. S. Kobayashi Y. Howe G. A. (2006). Identification of a peroxisomal acyl-activating enzyme involved in the biosynthesis of jasmonic acid in *Arabidopsis*. *J. Biol. Chem.* 281 33511–33520. 10.1074/jbc.M607854200 16963437

[B39] Koonin E. V. (2005). Orthologs, paralogs, and evolutionary genomics. *Annu. Rev. Genet.* 39 309–338. 10.1146/annurev.genet.39.073003.114725 16285863

[B40] Ku H. M. Vision T. Liu J. P. Tanksley S. D. (2000). Comparing sequenced segments of the tomato and *Arabidopsis* genomes: large-scale duplication followed by selective gene loss creates a network of synteny. *Proc. Natl. Acad. Sci. U.S.A.* 97 9121–9126. 10.1073/pnas.160271297 10908680 PMC16832

[B41] Kumar S. Stecher G. Tamura K. (2016). MEGA7: molecular evolutionary genetics analysis version 7.0 for bigger datasets. *Mol. Biol. Evol.* 33 1870–1874. 10.1093/molbev/msw054 27004904 PMC8210823

[B42] Lavhale S. G. Kalunke R. M. Giri A. P. (2018). Structural, functional and evolutionary diversity of 4-coumarate-CoA ligase in plants. *Planta* 248 1063–1078. 10.1007/s00425-018-2965-z 30078075

[B43] Lee D. Douglas C. J. (1996). Two divergent members of a tobacco 4-coumarate:coenzyme A ligase (4CL) gene family (cDNA structure, gene inheritance and expression, and properties of recombinant proteins). *Plant Physiol.* 112 193–205. 10.1104/pp.112.1.193 8819324 PMC157938

[B44] Lescot M. Déhais P. Thijs G. Marchal K. Moreau Y. Van de Peer Y. (2002). PlantCARE, a database of plant cis-acting regulatory elements and a portal to tools for in silico analysis of promoter sequences. *Nucleic Acids Res.* 30 325–327. 11752327 10.1093/nar/30.1.325 PMC99092

[B45] Letunic I. Bork P. (2018). 20 years of the SMART protein domain annotation resource. *Nucleic Acids Res.* 46 493–496. 10.1093/nar/gkx922 29040681 PMC5753352

[B46] Licausi F. Kosmacz M. Weits D. A. Giuntoli B. Giorgi F. M. Voesenek L. A. C. J. (2011). Oxygen sensing in plants is mediated by an N-end rule pathway for protein destabilization. *Nature* 479 419–422. 10.1038/nature10536 22020282

[B47] Liu J. P. Piñeros M. A. Kochian L. V. (2014). The role of aluminum sensing and signaling in plant aluminum resistance. *J. Integr. Plant Biol.* 56 221–230. 10.1111/jipb.12162 24417891

[B48] Liu M. Xu J. Lou H. Fan W. Yang J. Zheng S. (2016). Characterization of VuMATE1 expression in response to iron nutrition and aluminum stress reveals adaptation of rice bean (*Vigna umbellata*) to acid soils through Cis regulation. *Front. Plant Sci.* 7:511. 10.3389/fpls.2016.00511 27148333 PMC4835453

[B49] Liu S. Zhao L. Liao Y. Luo Z. Wang H. Wang P. (2020). Dysfunction of the 4-coumarate:coenzyme A ligase 4CL4 impacts aluminum resistance and lignin accumulation in rice. *Plant J.* 104 1233–1250. 10.1111/tpj.14995 32989851

[B50] Lou H. Q. Fan W. Xu J. M. Gong Y. L. Jin J. F. Chen W. W. (2016a). An oxalyl-CoA synthetase is involved in oxalate degradation and aluminum tolerance. *Plant Physiol.* 172 1679–1690. 10.1104/pp.16.01106 27650448 PMC5100784

[B51] Lou H. Q. Gong Y. L. Fan W. Xu J. M. Liu Y. Cao M. J. (2016b). A formate dehydrogenase confers tolerance to aluminum and low pH. *Plant Physiol.* 171 294–305. 10.1104/pp.16.01105 27021188 PMC4854670

[B52] Lü S. Y. Song T. Kosma D. K. Parsons E. P. Rowland O. Jenks M. A. (2009). *Arabidopsis* CER8 encodes LONG-CHAIN ACYL-COA SYNTHETASE 1 (LACS1) that has overlapping functions with LACS2 in plant wax and cutin synthesis. *Plant J.* 59 553–564. 10.1111/j.1365-313x.2009.03892.x 19392700

[B53] Ma J. F. (2007). Syndrome of aluminum toxicity and diversity of aluminum resistance in higher plants. *Int. Rev. Cytol.* 264 225–252. 10.1016/S0074-7696(07)64005-4 17964924

[B54] Maron L. G. Guimarães C. T. Kirst M. Albert P. S. Birchler J. A. Bradbury P. J. (2013). Aluminum tolerance in maize is associated with higher MATE1 gene copy number. *Proc. Natl. Acad. Sci. U.S.A.* 110 5241–5246. 10.1073/pnas.1220766110 23479633 PMC3612656

[B55] Molano-Flores B. (2001). Herbivory and calcium concentrations affect calcium oxalate crystal formation in Leaves of Sida (Malvaceae). *Ann. Bot.* 88 387–391. 10.1006/anbo.2001.1492

[B56] Palmieri F. Estoppey A. House G. L. Lohberger A. Bindschedler S. Chain P. S. G. (2019). Oxalic acid, a molecule at the crossroads of bacterial-fungal interactions. *Adv. Appl. Microbiol.* 106 49–77. 10.1016/bs.aambs.2018.10.001 30798804

[B57] Ryan P. R. Tyerman S. D. Sasaki T. Furuichi T. Yamamoto Y. Zhang W. H. (2011). The identification of aluminium-resistance genes provides opportunities for enhancing crop production on acid soils. *J. Exp. Bot.* 62 9–20. 10.1093/jxb/erq272 20847099

[B58] Sato S. Tabata S. Hirakawa H. Asamizu E. Shirasawa K. Isobe S. (2012). The tomato genome sequence provides insights into fleshy fruit evolution. *Nature* 485 635–641. 10.1038/nature11119 22660326 PMC3378239

[B59] Schmidt R. R. van Dongen J. T. (2019). The ACBP1-RAP2.12 signalling hub: a new perspective on integrative signalling during hypoxia in plants. *Plant Signal. Behav.* 14:e1651184. 10.1080/15592324.2019.1651184 31397636 PMC6768276

[B60] Schmidt R. R. Fulda M. Paul M. V. Anders M. Plum F. Weits D. A. (2018). Low-oxygen response is triggered by an ATP-dependent shift in oleoyl-CoA in *Arabidopsis*. *Proc. Natl. Acad. Sci. U.S.A.* 115 E12101–E12110. 10.1073/pnas.1809429115 30509981 PMC6304976

[B61] Schnurr J. Shockey J. Browse J. (2004). The acyl-CoA synthetase encoded by LACS2 is essential for normal cuticle development in *Arabidopsis*. *Plant Cell* 16 629–642. 10.1105/tpc.017608 14973169 PMC385277

[B62] Shang H. H. Li W. Zou C. S. Yuan Y. L. (2013). Analyses of the NAC transcription factor gene family in *Gossypium raimondii* Ulbr.: chromosomal location, structure, phylogeny, and expression patterns. *J. Integr. Plant Biol.* 55 663–676. 10.1111/jipb.12085 23756542

[B63] Shinde B. A. Dholakia B. B. Hussain K. Panda S. Meir S. Rogachev I. (2017). Dynamic metabolic reprogramming of steroidal glycol-alkaloid and phenylpropanoid biosynthesis may impart early blight resistance in wild tomato (*Solanum arcanum* Peralta). *Plant Mol. Biol.* 95 411–423. 10.1007/s11103-017-0660-2 28980117

[B64] Shockey J. M. Fulda M. S. Browse J. (2003). *Arabidopsis* contains a large superfamily of acyl-activating enzymes. phylogenetic and biochemical analysis reveals a new class of acyl-coenzyme A synthetases. *Plant Physiol.* 132 1065–1076. 10.1104/pp.103.020552 12805634 PMC167044

[B65] Shockey J. M. Fulda M. S. Browse J. A. (2002). *Arabidopsis* contains nine long-chain acyl-coenzyme A synthetase genes that participate in fatty acid and glycerolipid metabolism. *Plant Physiol.* 129 1710–1722. 10.1104/pp.003269 12177484 PMC166759

[B66] Shockey J. Browse J. (2011). Genome-level and biochemical diversity of the acyl-activating enzyme superfamily in plants: biochemistry and evolution of plant AAE proteins. *Plant J.* 66 143–160. 10.1111/j.1365-313X.2011.04512.x 21443629

[B67] Soltani B. M. Ehlting J. Hamberger B. Douglas C. J. (2006). Multiple cis-regulatory elements regulate distinct and complex patterns of developmental and wound-induced expression of *Arabidopsis thaliana* 4CL gene family members. *Planta* 224 1226–1238. 10.1007/s00425-006-0296-y 16738863

[B68] Staswick P. E. Tiryaki I. Rowe M. L. (2002). Jasmonate response locus JAR1 and several related *Arabidopsis* genes encode enzymes of the firefly luciferase superfamily that show activity on jasmonic, salicylic, and indole-3-acetic acids in an assay for adenylation. *Plant Cell* 14 1405–1415. 10.1105/tpc.000885 12084835 PMC150788

[B69] Sun H. Li Y. Feng S. Zou W. Guo K. Fan C. (2013). Analysis of five rice 4-coumarate:coenzyme A ligase enzyme activity and stress response for potential roles in lignin and flavonoid biosynthesis in rice. *Biochem. Biophys. Res. Commun.* 430 1151–1156. 10.1016/j.bbrc.2012.12.019 23246835

[B70] Tang D. Z. Simonich M. T. Innes R. W. (2007). Mutations in LACS2, a long-chain acyl-coenzyme A synthetase, enhance susceptibility to avirulent *Pseudomonas syringae* but confer resistance to *Botrytis cinerea* in Arabidopsis. *Plant Physiol.* 144 1093–1103. 10.1104/pp.106.094318 17434992 PMC1914183

[B71] Tsutsui T. Yamaji N. Feng Ma J. (2011). Identification of a Cis-Acting element of ART1, a C2H2-Type zinc-finger transcription factor for aluminum tolerance in rice. *Plant Physiol.* 156 925–931. 10.1104/pp.111.175802 21502187 PMC3177286

[B72] Veitia R. A. (2005). Paralogs in polyploids: one for all and all for one? *Plant Cell* 17 4–11. 10.1105/tpc.104.170130 15632052 PMC544485

[B73] von Uexküll H. R. Mutert E. (1995). Global extent, development and economic impact of acid soils. *Plant Soil* 171 1–15. 10.1007/BF00009558

[B74] Wang G. D. Zhang S. Ma X. C. Wang Y. Kong F. Y. Meng Q. W. (2016). A stress-associated NAC transcription factor (SlNAC35) from tomato plays a positive role in biotic and abiotic stresses. *Physiol. Plant.* 158 45–64. 10.1111/ppl.12444 26991441

[B75] Wang X. Y. Paterson A. H. (2011). Gene conversion in angiosperm genomes with an emphasis on genes duplicated by polyploidization. *Genes* 2 1–20. 10.3390/genes2010001 24710136 PMC3924838

[B76] Wang Y. P. Tang H. B. Debarry J. D. Tan X. Li J. Wang X. Y. (2012). MCScanX: a toolkit for detection and evolutionary analysis of gene synteny and collinearity. *Nucleic Acids Res.* 40 49–63. 10.1093/nar/gkr1293 22217600 PMC3326336

[B77] Weng H. Molina I. Shockey J. Browse J. (2010). Organ fusion and defective cuticle function in a lacs1 lacs2 double mutant of *Arabidopsis*. *Planta* 231 1089–1100. 10.1007/s00425-010-1110-4 20237894

[B78] Xian P. Q. Cai Z. D. Cheng Y. B. Lin R. B. Lian T. X. Ma Q. B. (2020). Wild soybean oxalyl-CoA synthetase degrades oxalate and affects the tolerance to cadmium and aluminum stresses. *Int. J. Mol. Sci.* 21:8869. 10.3390/ijms21228869 33238600 PMC7700444

[B79] Xie L. J. Tan W. J. Yang Y. C. Tan Y. F. Zhou Y. Zhou D. M. (2020). Long-chain acyl-CoA synthetase LACS2 contributes to submergence tolerance by modulating cuticle permeability in *Arabidopsis*. *Plants* 9:262. 10.3390/plants9020262 32085442 PMC7076686

[B80] Xie T. Chen C. J. Li C. H. Liu J. R. Liu C. Y. He Y. H. (2018). Genome-wide investigation of WRKY gene family in pineapple: evolution and expression profiles during development and stress. *BMC Genomics* 19:490. 10.1186/s12864-018-4880-x 29940851 PMC6019807

[B81] Yang J. L. Fan W. Zheng S. J. (2019). Mechanisms and regulation of aluminum-induced secretion of organic acid anions from plant roots. *J. Zhejiang Univ. Sci. B* 20 513–527. 10.1631/jzus.b1900188 31090277 PMC6568218

[B82] Yang X. J. Liang W. Q. Chen M. J. Zhang D. B. Zhao X. X. Shi J. X. (2017). Rice fatty acyl-CoA synthetase OsACOS12 is required for tapetum programmed cell death and male fertility. *Planta* 246 105–122. 10.1007/s00425-017-2691-y 28382520

[B83] Yu G. C. Smith D. K. Zhu H. C. Guan Y. Lam T. T. Y. (2017). GGTREE: an r package for visualization and annotation of phylogenetic trees with their covariates and other associated data. *Methods Ecol. Evol.* 8 28–36. 10.1111/2041-210x.12628

[B84] Zhao H. Y. Kosma D. K. Lü S. Y. (2021). Functional role of long-chain acyl-CoA synthetases in plant development and stress responses. *Front. Plant Sci.* 12:640996. 10.3389/fpls.2021.640996 33828572 PMC8019973

[B85] Zhao L. F. Haslam T. M. Sonntag A. Molina I. Kunst L. (2019). Functional overlap of long-chain acyl-CoA synthetases in *Arabidopsis*. *Plant Cell Physiol.* 60 1041–1054. 10.1093/pcp/pcz019 30715495

[B86] Zhou M. Q. Thompson W. A. Tang W. (2020). The *Arabidopsis* AtAAE13.1 gene enhances salt stress tolerance in angiosperms and gymnosperm plant cells. *Vitro Cell. Dev. Biol. Plant* 56 750–764. 10.1007/s11627-020-10083-y

