# Supplementary material for: Genome-Wide Identification and Gene Expression Analysis of Acyl-Activating Enzymes Superfamily in Tomato (Solanum lycopersicum) Under Aluminum Stress
Source: Front Plant Sci. 2021 Dec 2;12:754147. doi: 10.3389/fpls.2021.754147 (PMC8674732; doi:10.3389/fpls.2021.754147)
Supplement: Supplementary file 1 [file Data_Sheet_1.zip › Supplementary Figure 1.DOCX]

Supplementary Material

# Supplementary Figure


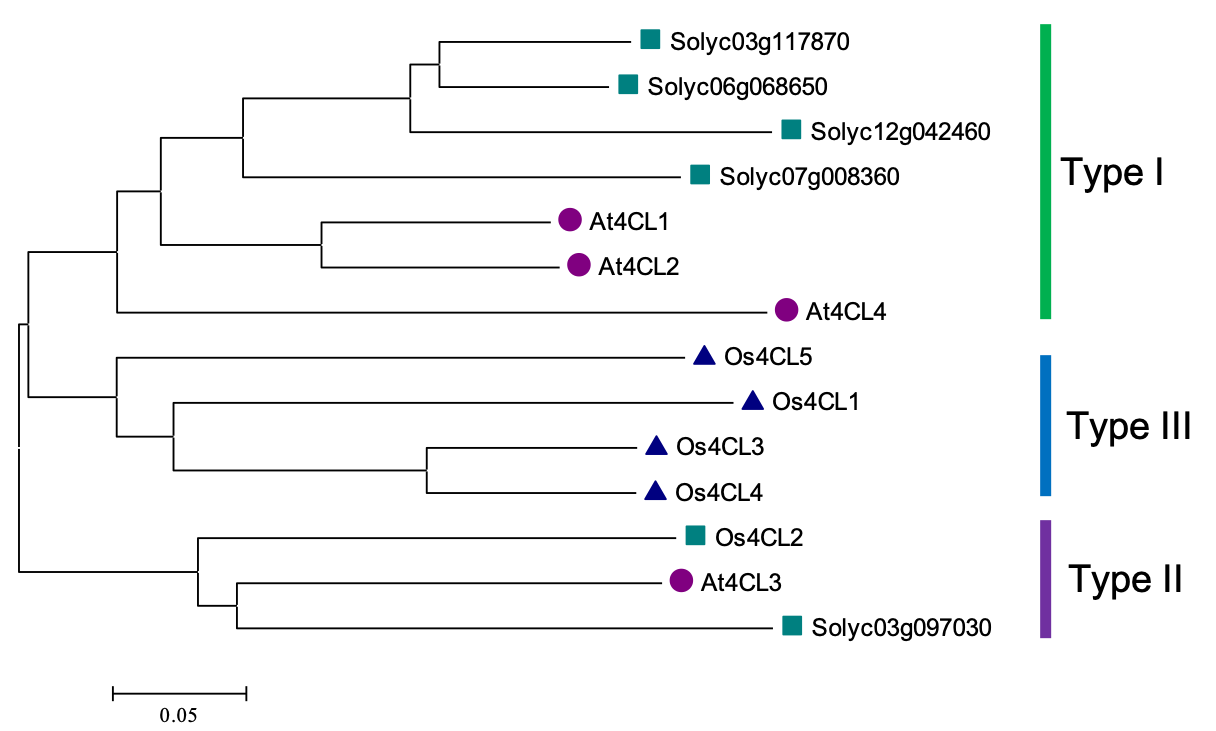


**Supplementary Figure 1.** Unrooted phylogenetic tree of 4CL isoforms from rice, tomato and Arabidopsis was constructed with bootstrap values after 1,000 trials. Plant species are as follows: green rectangles, tomato; purple circles, Arabidopsis; blue triangles, rice.
